# Germ Cell Neoplasia *in situ* (GCNIS) in Testis-Sparing Surgery (TSS) for Small Testicular Masses (STMs)

**DOI:** 10.3389/fendo.2019.00512

**Published:** 2019-08-07

**Authors:** Francesco Pierconti, Maurizio Martini, Giuseppe Grande, Luigi M. Larocca, Emilio Sacco, Dario Pugliese, Gaetano Gulino, Pier F. Bassi, Domenico Milardi, Alfredo Pontecorvi

**Affiliations:** ^1^Institute of Pathology, Fondazione Policlinico Gemelli IRCCS, Università Cattolica del Sacro Cuore, Rome, Italy; ^2^Division of Endocrinology, Istituto Scientifico Internazionale “Paolo VI”, Fondazione Policlinico Gemelli IRCCS, Università Cattolica del Sacro Cuore, Rome, Italy; ^3^Institute of Urology, Fondazione Policlinico Gemelli IRCCS, Università Cattolica del Sacro Cuore, Rome, Italy

**Keywords:** testis, small testicular mass, seminoma, intratubular germ cell neoplasia, PLAP

## Abstract

**Purpose:** The testis-sparing surgery (TSS) is surgical technique accepted for small testicular masses (STMs). Frozen section examination (FSE) is an essential assessment at the time of TSS. The aim of this study is to measure the maximum distance of the foci of ITGCN from STMs.

**Methods:** In our hospital between June 2010 and October 2017 a total of 68 patients with STM underwent a TSS. All the testis specimens were totally embedded and processed via the whole-mount method and a diagnosis of germ cell tumor with GCNIS were made. The distance between STMs and GCNIS were calculated by two pathologists directly on the slides considering for the third dimension the number of the paraffin blocks in which the foci of GCNIS were found.

**Results:** The STMs were classic seminoma in 62 out 68 cases, embryonal carcinoma in 4 cases, while in 2 case a diagnose of mixed germ cell tumor were made. The size of the STMs was between 0.5 and 2 cm and the foci of GCNIS were observed in seminiferous tubules very closed to SMTs or as skip lesions in the surrounding testicular parenchyma, dispersed in normal testis. In 48 out of 68 cases (70.5%) foci of GCNIS were at the distance from SMTS of 1.5 cm or below and in 60 out of 68 cases (88%) at the distance of 2 cm or below The distance of GCNIS from the STMs was not related to the histological subtype of the germ cell tumor, while there is a linear correlation between size of the STMs and the distance of foci of GCNIS (*p* = 0.0105; *r* = 0.9167).

**Conclusion:** Our data showed that foci of ITGCN were not observed beyond 2.5 cm from the STM. In particular we demonstrated that exist a linear correlation between size of STMs and distance of the foci of GCNIS from STMs (*p* = 0.0105; *r* = 0.9167). In conclusion mapping the tissue around the tumor not randomly but in targeted areas could reduce the false negative biopsies of the testis with GCNIS, increasing the radicality of the TSS procedure.

## Introduction

Malignant germ cell tumors appear clinically as palpable masses and radical orchiectomy is considered the standard surgical treatment for these lesions (1). The recent use of high-frequency ultrasonography has led to an increase in detection of incidental small testicular masses (STMs) defined as non-palpable, <25 mm in diameter, intrascrotal masses (2). The testis-sparing surgery (TSS) is an accepted surgical technique for STMs. Since most STMs are benign, unnecessary orchiectomy is to be considered too “costly” from a hormonal and reproductive point of view (3–5). Organ-sparing surgery has numerous advantages from an endocrine point of view in avoiding hypogonadism (6). Orchiectomy in fact causes a reduction in testosterone levels, which in turns represents a risk factor for cardiovascular diseases (7), osteoporosis (8), and lower urinary tract syndrome (LUTS) (9). As a consequence, patients who underwent orchiectomy often require life-long androgen replacement therapy and have a higher risk of low bone mineral density (10) and LUTS (11). Furthermore, preservation of fertility is another issue in young patients affected by testicular tumors. Moreover, it has been reported that unilateral orchiectomy has disruptive effects on overall spermatogenesis (4). Liu et al. reported no significant changes in terms of sperm concentrations and motility in 11 patients with TSS history for benign testicular tumors (12).

The indications for TSS are still controversial, especially for patients with normal contralateral testis. According to the German Cancer Study Group, TSS can be considered only for selected patients with malignant tumors in solitary testis or with a bilateral tumor with a lesion diameter <2 cm and no invasion of rete testis, with normal preoperative serum (LH) levels (13).

Moreover in 2011, the update of the EAU Guidelines considered TSS as an alternative surgical treatment only for patients with synchronous bilateral testicular tumors, metachronous contralateral tumors or for lesions in patients with solitary testis, and normal preoperative testosterone levels if the volume of the tumors represents <30% of the testicular volume.

To avoid secondary surgery, the urologist needs to know the quality of the resected small tumor and the surrounding tissue. The diameter of the mass is an important parameter for TSS; several studies demonstrated that in the case of non-palpable, symptomatic masses with a diameter of <2 cm, TSS represents the best surgical management because the prevalence of benign histology is ~80% (14–19).

Multiple biopsies of the surrounding tissue, in order to evaluate the presence of microscopic areas of tumor infiltration, is performed after the enucleation of the mass. The presence of foci of GCNIS in the multiple biopsies surrounding the small testicular mass (STM), at frozen section examination (FSE), could help the pathologist discern benign from malignant neoplasms, when a non-conclusive diagnosis of STMs is made. In fact, the presence of GCNIS near the mass seems sufficient for the diagnosis of malignant testicular germ cell tumors (TGCTs) (20–25).

In case of the diagnosis of TGCTs, adjuvant approaches such as radiotherapy, chemotherapy (one cycle of carbolatin AUC7 in case of seminoma) or surveillance must be considered.

The goal of our study is to define the maximum distance of the expected presence of foci of GCNIS from malignant STMs, in order to provide the surgeon with significant information during the intraoperative procedures of TSS.

## Materials and Methods

Sixty-eight patients with a STM <2 cm and with a volume <30% of the testicular volume, underwent TSS in our hospital between June 2010 and October 2017. All patients had a testicular mass <2 cm at inspection and a scrotal high frequency ultrasound (US); no physical or radiological signs of malignancy or metastases were observed.

During surgical procedures the FSE of the STMS and multiple biopsies of the surrounding testicular tissue were performed. An operative ultrasound was applied to permit the surgeon to measure and perform the biopsies at the right distance.

As a result of the diagnosis, a traditional radical orchiectomy was performed in case of diagnosis of GCT and/or GCNIS identification in FSE, in order to ensure the best oncological result.

All the diagnosis made on frozen section biopsies were confirmed at histological examination after immune-histochemical staining with PLAP and CD117.

All testis specimens were totally embedded and processed via the whole-mount method and a final pathology reports of germ cell tumors with GCNIS were made (26, 27). For the diagnosis of the GCNIS we isolated the areas with histological features of GCNIS in the whole mount section. A confirmative immune-histochemical staining with PLAP and CD117 was performed.

The distance between STMs and GCNIS was calculated by two pathologists (LML and FP) on the whole mount section, directly on the slides, considering the third dimension for the number of paraffin blocks in which the foci of GCNIS were found. Each paraffin block was 5 mm in thickness.

For the immune-histochemical studies, the avidin-biotin-peroxidase complex method was performed on the paraffin sections, applying a technical procedure previously reported, using a commercially available kit (Dako LSAB2, Dakopatts, Glostrup, Denmark) and the following commercially available monoclonal antibodies: PLAP, CD117 (28, 29).

Statistical analysis was performed using GraphPad Prism (version 5, La Jolla, CA) or MedCalc (version 10.2, Ostend, Belgium) software. Continuous variables normally distributed were reported as the mean and standard deviation (SD). A comparison of continuous variables normally distributed was performed using the Student's *t-*test (U-paired or Mann-Whitney *t*-test). A comparison of categorical variables between the two groups was performed by the Chi-square statistic or using the Fisher exact test when appropriate. All *p*-values are considered statistically significant when *p* < 0.05.

## Results

**Figure 1:**
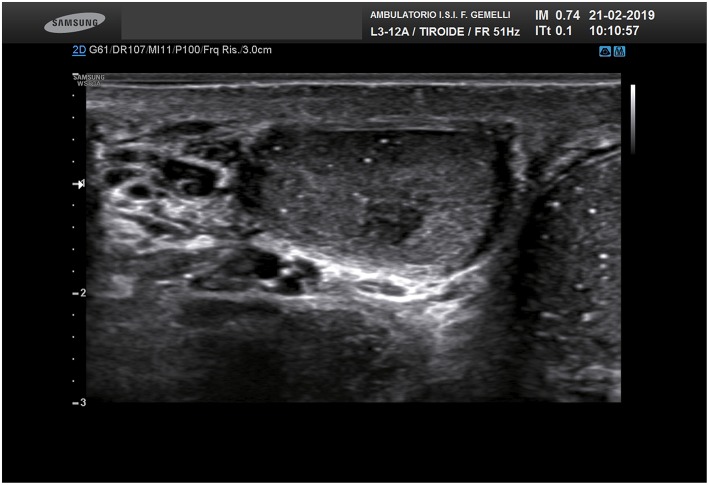
Ultrasound image of STM.

The main clinical features of the patients, including the main risk factors for TGCTs, are reported in Table 1. Figure 1 shows an ultrasound image explicative of a STM.

**Table 1:** Main clinical features of the patients (continuous variables are expressed as mean ± DS).

		**Min-max**
NST (*n*.)	6/68	
ST (*n*.)	62/68	
Age (years)	37 ± 11	(25-60)
Tumor size (mm)	13.7 ± 4.3	(5-20)
Testis volume (ml)	20.8 ± 8.5	(5-30)
FSE distance (mm)	16.1 ± 3.4	(10-24)
Cryptorchidism	27/68	
Infertility	21/68	
Chemical exposition	1/68	

Classic seminoma was found in 62 out of 68 cases with STMs, embryonal carcinoma was found in four, while a mixed germ cell tumor was found in two (seminoma, embryonal carcinoma, and yolk sac tumor). No histological signs of neoplastic invasion of rete testis or necrosis were observed.

The size of the STMs was between 0.5 and 2 cm and the foci of GCNIS were observed in seminiferous tubules very close to SMTs or as skip lesions in the surrounding testicular parenchyma. In 24 cases, the foci of GCNIS were at a distance of 1.5 cm or below from SMTS and in 31 cases at a distance of 2 cm or below. GCNIS was not detected in 13 FSE biopsies.

**Figure 2:**
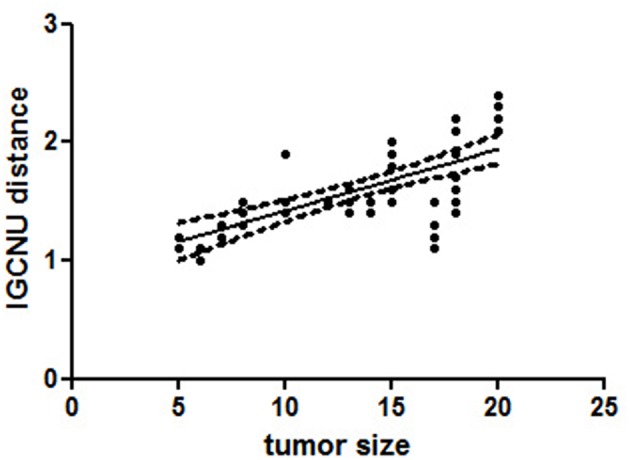
A linear regression analysis performed to evaluate the significant association between different variables [tumor size and distance of foci germ cell neoplasia *in situ* (GCNIS) showed a linear correlation between size of the small testicular masses (STMs) and the distance of foci of GCNIS (*p* = 0.0105; r = 0.9167)]. All *p*-values are considered statistically significant when *p* < 0.05.

The distance of GCNIS from the STMs was not related to the histological subtype of the germ cell tumor, while there was a linear correlation between the size of the STMs and the distance of foci of ITGCN (*p* = 0.0105; *r* = 0.9167; Figure 2).

**Figure 3:**
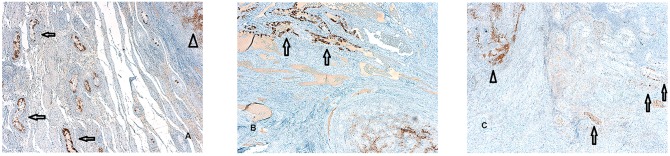
**(A–C)** Foci of germ cell neoplasia *in situ* (GCNIS) identified by PLAP immunostaining surrounding the small testicular mass (STM) diagnosed as seminoma. A focal rather than random distribution of the GCNIS in testicular tissue adjacent to TGCTS was evident (PLAP immunostaining magnification 5X). This foci of GCNIS (arrows) are separated form the STM (arrows head) by tubular testis without the sign of intratubular germ cell neplasia and in all the cases examined,foci of GCNIS were not observed beyond 2,5 cm from the STM.

In detail, foci of GCNIS have been found within 2 cm from the STMs with a diameter ≤ 1.5 cm. GCNIS have been found up to 2.5 cm from the neoplastic lesion, when tumor size ranged from 1.5 cm up to 2 cm, We did not observe foci of GCNIS beyond 2.5 cm from the STMs (Figures 3A–C).

## Discussion

The use of an ultrasound in the primary evaluation of patients with infertility and with local scrotal symptoms has led to high incidence in the early detection of small, mostly benign, testicular masses (3). According to the literature, testis sparing surgery is a safe and feasible procedure for patients presenting a benign small testis mass. It has been demonstrated that more than two thirds of asymptomatic testicular masses <2 cm are pathologically benign (13, 16, 17, 19). Although TSS is a controversial approach (30), it is justified in highly selected clinical scenarios.

In our study we performed TSS and FSE in a population of selected patients with STMs. When a diagnosis of malignant tumors was made after TSS, the orchiectomy was performed in our study. However, several papers demonstrated that the surveillance of STMs is an option along with testis sparing surgery (30).

In our study, FSE of the primary lesion and the surrounding tissue was performed to determine the nature of the testicular mass and to evaluate the radicality of the surgical procedure (3, 31). FSE has gained increasing accuracy, after initial skepticism about its diagnostic power. FSE represents a useful tool, especially with the aim to discriminate between benign or malignant testicular neoplasms when a histological diagnosis of STM is not conclusive (16, 20–22, 25). Sometimes in fact, the FSE of the STM is unable to discriminate between benign and malignant tumors, especially when the STM is very small, when the material for the FSE is poor or when the tissue resected is affected by artifacts, caused by technical frozen procedures (18, 32, 33). In these cases, identifying foci of GCNIS near the STM may help pathologists to classify STM as a germ cell tumor. In fact, previous data have been reported demonstrating that foci of GCNIS were found in up to 82% of tissues surrounding a germ cell tumor and that the risk of progression to invasive malignancy is 50 and 70% at 5- and 7-years follow-ups, respectively (13, 34).

FSE in surrounding tissue is a very accurate procedure. It has been reported in fact that the false-negative biopsies for the diagnosis of GCNIS has been found in only 0.5% of the biopsies (35). The majority of false negative biopsies are caused by sampling errors when a biopsy is not taken from a representative area.

Our data showed that foci of GCNIS have not been observed beyond 2.5 cm from the STM. To provide detail, we demonstrated the linear correlation between the size of STMs and the distance of the GCNIS foci from STMs. For small masses with a diameter up to 1.5 cm, GCNIS was observed up to 2 cm from the tumor. When the tumor diameter was more than 1.5 cm, GCNIS foci was detected up to 2.5 cm from the tumor. These results are in accordance with GCNIS-mapping studies that reported a focal rather than random distribution of the GCNIS in testicular tissue adjacent to TGCTs (36, 37).

Despite the limitations of this study, since it is a retrospective study analyzing TSS with targeted biopsies, it is the first study providing data on the correct distance to perform FSE in TSS from. Further studies are needed to provide confirmation in total orchiectomies and namely to analyze the linear correlation between the size of GCT and the distance from GNIS in orchiectomy samples. These studies will provide clearer evidence to support partial orchiectomy.

Moreover, targeted testicular biopsies performed at the right distance from STMs and measured by intraoperative US, might reduce the false negative rates in the diagnosis of GCNIS, thus supporting pathologists in detecting clinical situations at risk for malignant TGCTs.

The knowledge of the distance, from STM beyond which we did not find GCNIS, could help surgeons during intraoperative procedures, suggesting the best site at which to perform the testicular biopsies for the FSE.

In conclusion, the histological characterization by FES of the tissue around tumors in targeted areas could reduce false negative biopsies of testis with GCNIS. This approach may improve the efficacy of radical excision through the TSS procedure, reducing the risk of leaving residual cancer during TSS and subsequently of the disease recurrence.

## Ethics Statement

All the procedures described in this paper are included in the consensus statement of our hospital that the patients signed when a surgical approach is recommended. The FSE of the STMS, the multiple biopsies of the surrounding testicular tissue and the processing of the neoplastic nodule via the whole-mount method are routinary processes in our department of pathology as well as the immunohistochemical staining with PLAP and CD117 performed in order to make a correct histopathological diagnosis. For this reasons, we believe that an ethical review process was not required for this study.

## Author Contributions

All authors listed have made a substantial, direct and intellectual contribution to the work, and approved it for publication.

### Conflict of Interest Statement

The authors declare that the research was conducted in the absence of any commercial or financial relationships that could be construed as a potential conflict of interest.
